# Delta neutrophil index as a predictor of disease severity, surgical outcomes, and mortality rates in gastrointestinal diseases: Rationale for a meta-analysis of diagnostic test accuracy

**DOI:** 10.1097/MD.0000000000017059

**Published:** 2019-08-30

**Authors:** Jae Ho Choi, Chang Seok Bang, Jae Jun Lee, Gwang Ho Baik

**Affiliations:** a Institute of New Frontier Research; b Department of Internal Medicine, Hallym University College of Medicine; c Institute for Liver and Digestive Diseases, Hallym University; d Department of Anesthesiology and Pain Medicine, Hallym University College of Medicine, Chuncheon, Korea.

**Keywords:** delta neutrophil index, diagnosis, prognosis, severity, surgery

## Abstract

**Background:**

Delta neutrophil index (DNI) is the ratio of the number of immature granulocytes and the total neutrophil count in peripheral circulation. DNI precedes changes in white blood cell or neutrophil counts due to the course of granular leukocyte differentiation in infectious and inflammatory conditions, beginning with immature granulocyte formation. The role of DNI as a biomarker of various infectious or inflammatory conditions has been reported. However, no studies explored the potential role of DNI as an initial biomarker for predicting disease severity, surgical outcomes, and mortality rates of gastrointestinal diseases with pooled diagnostic test accuracy. This study aims to provide evidence that DNI is a predictor of disease severity, surgical outcomes, and mortality rates in patients with gastrointestinal diseases in emergency medical departments.

**Methods:**

MEDLINE, EMBASE, and the Cochrane Library will be searched using common keywords (inception to July 2019) by 2 independent evaluators. Inclusion criteria will be patients with gastrointestinal diseases, DNI measurements performed in the emergency department, indices of diagnostic performance (sensitivity, specificity, predictive values, and likelihood ratios) of DNI for predicting severity, surgical outcomes, and mortality rate of gastrointestinal diseases. True and false positives and negatives will be calculated based on the diagnostic indices of each study. All types of study designs with full-text literature written in English will be included. Risk of bias will be assessed using the Quality Assessment of Diagnostic Accuracy Studies-2 (QUADAS-2) tool. Descriptive data synthesis will be conducted and quantitative synthesis (bivariate and hierarchical summary receiver operating characteristic model) will be performed if the included studies are sufficiently homogenous. Meta-regression, sensitivity analysis, publication bias, and Fagan nomogram will be analyzed and described.

**Results:**

The pooled synthesis of the diagnostic performance of various gastrointestinal diseases with different cut-off values for DNI may limit the interpretation of uniform diagnostic validity. The authors will contact the corresponding authors for the missing values, requesting the original data in each study. However, if there are no responses from these authors, these studies will be excluded.

**Conclusion:**

This study will provide diagnostic validity of DNI as an initial marker for the prediction of severity, surgery, and mortality of gastrointestinal diseases.

## 1 Introduction

Gastrointestinal diseases are a significant source of morbidity and impose a substantial economic burden worldwide. Approximately 13% of the total annual medical expenditures in Korea are attributable to gastrointestinal diseases,^[1]^ and a total of $135.9 billion was spent in the United States. This is a greater financial expenditure than that for many other common and important diseases, such as cardiac diseases, trauma-related conditions, and psychiatric disorders.^[2]^ Furthermore, these expenditures are likely to continue to increase in the United States,^[2]^ and the overall emergency department visits for gastrointestinal diseases have been increasing.^[3]^ The annual hospitalization burden for emergency gastrointestinal conditions in the United States is estimated at 2.6 million patients, and nearly one-third of these patients require surgery.^[4]^ Further, the annual incidence of hospitalization for gastrointestinal diseases has also increased in Hong Kong, Korea.^[5]^ As such, while acute appendicitis, cholecystitis, and intestinal obstruction are common, they represent a significant toll to health care systems.^[4]^

Clinical prediction models have been actively developed, which provided insight into patients who are at an increased risk of surgery-related complications, the most severe etiologies, and death.^[4]^ However, these prediction models require rote memorization and complex calculations, and hence are generally not considered in nonsurgical clinical management.^[4]^

Delta neutrophil index (DNI) is a calculated parameter that measures the ratio of number of immature granulocytes to the total neutrophil count in peripheral circulation.^[6]^ DNI precedes changes in white blood cell or neutrophil counts. This is primarily a result of granular leukocyte differentiation in infectious and inflammatory conditions commencing with the formation of immature granulocytes.^[7]^ DNI is estimated by subtracting the fraction of mature polymorphonuclear leukocytes from the sum of myeloperoxidase-reactive cells.^[6,8]^ As a result, it is a biomarker that reflects the number of immature neutrophils in peripheral circulation.^[8]^

Previous studies have reported the potential role of DNI as a biomarker of various infectious or inflammatory conditions. The measurement of the DNI is reproducible, rapid, and accurate.^[8,9]^ However, there have been no studies which have explored the potential role of DNI as an initial biomarker for the prediction of disease severity, surgical outcomes, and mortality rates of gastrointestinal diseases in emergency departments.

The current study aims to provide evidence that DNI is an initial biomarker for the prediction of disease severity, surgical outcomes, and mortality rates of gastrointestinal diseases presented in emergency departments.

## 2 Methods

This systematic review and meta-analysis fully adheres to the principles of the Preferred Reporting Items for Systematic reviews and Meta-Analyses (PRISMA-P) checklist.^[10]^ This study protocol was registered at PROSPERO (https://www.crd.york.ac.uk/prospero) on June 2019 (registration number, CRD42019136459) before the study was initiated. This study is exempt from approval by an institutional review board because it will only collect and synthesize data from previously published studies.^[11,12]^

### 2.1 Literature searching strategy

MEDLINE (through PubMed), EMBASE, and the Cochrane Central Register of Controlled Trials in the Cochrane Library will be queried using the common keywords “delta neutrophil index” (inception, July 2019) by 2 independent evaluators (JHC, and CSB). No search will be performed in the Medical Subject Heading or Emtree, and only the keyword “delta neutrophil index” will be used during all searches of electronic databases to maximize sensitivity. The abstracts of all identified studies will be reviewed to exclude irrelevant articles. Full-text reviews will be performed to determine whether the inclusion criteria were satisfied in the remaining studies, and the bibliographies of relevant articles will be reviewed to identify any additional publications. Disagreements between the evaluators will be resolved by discussion or consultation with a third evaluator (GHB).

### 2.2 Selection criteria

We will include studies that meet the following criteria: patients: patients with gastrointestinal diseases, intervention: studies where DNI measurements were made in the emergency department (in cases of admitted patients, the measurement performed at day 0 will be included), comparison: none, outcome: inclusion of diagnostic performance indices (sensitivity, specificity, positive predictive values, negative predictive values, likelihood ratios) of DNI for the prediction of disease severity, surgical outcomes, and mortality rates of gastrointestinal diseases (true positive (TP), false positive (FP), true negative (TN), and false negative (FN) values will be calculated based on the diagnostic indices in each study), study design: all types (case-control studies will be analyzed according to subgroups, as this can exaggerate the diagnostic test accuracy (DTA) due to selection bias), those studies with human subjects, and full-text publications written in English. Only studies that met all of the inclusion criteria will be selected and included in the analysis. The exclusion criteria are as follows: review articles, guidelines, consensus documents, or expert position papers, comments, letters, brief reports, proceedings, or protocol studies, publications with incomplete data, and meta-analysis articles. Studies meeting 1 or more of the exclusion criteria will be excluded from this analysis.

### 2.3 Methodological quality

The methodological quality of the included articles will be assessed using the Quality Assessment of Diagnostic Accuracy Studies-2 (QUADAS-2) tool.^[13]^ The QUADAS-2 tool contains 4 domains, including “patient selection,” “index test, ” “reference standard, ” and “flow and timing” (flow of the patients through the study and timing of the index tests and reference standard).^[13]^ The methodological quality assessment process consists of 4 phases: report the signaling review question, develop review-specific (tailoring) guidance, review the published flow diagram for the primary study, perform a judgment on the risk of bias and any concerns as to study applicability.^[13]^ Each domain is determined to exhibit high-, low-, or unclear risks of bias, and the first 3 domains are also determined to exhibit high-, low-, or unclear concerns about applicability.^[13]^ The results of the methodological quality assessment are described using a tabular presentation for each study. Two of the evaluators (JHC and CSB) will independently assess the methodological quality of all the included studies, and any disagreements between the evaluators will be resolved by discussion or consultation with a third evaluator (GHB).^[11,12]^

### 2.4 Data extraction and primary and modifier-based analyses

Two evaluators (JHC, and CSB) will independently use the same data form to collect the primary summary outcomes and modifiers in each study, and disagreements between the 2 evaluators will be resolved by discussion and/or consultation with a third evaluator (GHB).

DTA is the primary outcome of this study. We will calculate the TP (subjects with positive DNI who had a severe form of gastrointestinal disease, underwent surgery, or experienced in-hospital mortality due to gastrointestinal diseases), FP (subjects with a positive DNI who did not have a severe form of gastrointestinal diseases, underwent surgery, or experienced in-hospital mortality due to gastrointestinal diseases), FN (subjects with a negative DNI who had a severe form of gastrointestinal disease, underwent surgery, or experienced in-hospital mortality due to gastrointestinal diseases), and TN (subjects with a negative DNI who did not have a severe form of gastrointestinal disease, underwent surgery, or experienced in-hospital mortality due to gastrointestinal diseases) values of DNI for the prediction of disease severity, surgical outcomes, and mortality rates of gastrointestinal diseases based on the diagnostic performance indices of each study. These will include sensitivity, specificity, predictive values, and likelihood ratios using 2 × 2 tables, whenever possible, based on the original articles.

If only a portion of the data is presented, we will calculate the DTA using the following formulas: sensitivity, TP/(TP+FN); specificity, TN/(FP+TN); positive predictive value, TP/(TP+FP); negative predictive value, TN/(FN+TN); positive likelihood ratio, sensitivity/(1-specificity); negative likelihood ratio, (1-sensitivity)/specificity; accuracy, (TP+TN)/(TP+FP+FN+TN); diagnostic odds ratio, (TP × TN)/(FP × FN); standard error, (ln (upper confidence interval)—ln (lower confidence interval))/3.92 = √(1/TP+1/FP+1/FN+1/TN).^[11,12]^

The following data will also be extracted from each study, whenever possible: study design, age, sex, ethnicity of enrolled population, sample size, year published, diagnostic method, cut-off value of DNI, and form of gastrointestinal disease.

### 2.5 Statistical analysis

Narrative (descriptive) synthesis will be performed and quantitative synthesis (bivariate random model^[14]^ and hierarchical summary receiver operating characteristic (HSROC) model^[15]^ will be used if the included studies are sufficiently homogenous.

The quantitative synthesis and meta-analysis performed in this study will be conducted using Stata Statistical Software (version 13.0, relevant packages for analyses: metandi, midas, and mylabels, College Station, TX).

The common effect size (TP, FP, FN, and TN) will be extracted or calculated from each study and a pooled meta-analysis of the crude outcomes of each study with summary outcomes will be presented (i.e., the paired forest plot of pooled sensitivity or specificity with confidence region and prediction region using a bivariate model). An SROC curve will be generated and presented using an HSROC model. Heterogeneity across the studies will be determined by the correlation coefficient between logit transformed sensitivity and specificity by the bivariate model^[14]^ and the asymmetry parameter β (beta), where β = 0 corresponds to a symmetric receiver operating characteristic curve in which the diagnostic odds ratio does not vary along the curve according to the HSROC model.^[15,16]^ A positive correlation coefficient (greater than 0) and a β with a significant *P* value (*P* < .05) indicates heterogeneity between studies.^[15,16]^ Visual inspection of the SROC curve will also be performed in the determination of heterogeneity. Subgroup analysis and meta-regression using the modifiers identified during the systematic review will also be performed to confirm the robustness of the main result and to identify the reasons for heterogeneity in cases of quantitative synthesis. Publication bias will be evaluated using Deeks funnel plot asymmetry test.^[11,12]^

### 2.6 Ethics and dissemination

This protocol is a systematic review and meta-analysis for the diagnostic performance of DNI in the prediction of disease severity, surgical outcomes, and mortality rates of gastrointestinal diseases in emergency departments. This study protocol was registered at PROSPERO on June 2019 (registration number, CRD42019136459) prior to the study being initiated. This study is exempt from the approval of an institutional review board due to the characteristics of the study design (collecting and synthesizing data from previously published studies). Results from this study will be disseminated in peer-reviewed journals.

## 3 Discussion

This is the protocol of a systematic review and meta-analysis for the DNI as a predictor of disease severity, surgical outcomes, and mortality rates in patients with gastrointestinal diseases in emergency medical departments.

A recently published meta-analysis evaluated the role of DNI as a prognostic marker for mortality in adult patients with sepsis.^[8]^ However, this study primarily only reported on the association between elevated DNI and mortality in sepsis, and the diagnostic performance of DNI was calculated based only on 4 studies. Differential cut-off values found in each of the studies were not considered in that meta-analysis. An additional meta-analysis conducted previously evaluated the DTA of DNI as a diagnostic and prognostic marker of infection.^[9]^ The sensitivity and specificity for the diagnosis of infection were 0.67 and 0.94, respectively, and 0.70 and 0.78 for the prognosis of infection, respectively.^[9]^ However, only a bivariate model for DTA was assessed and the included studies were not limited to emergent conditions with gastrointestinal disorders. Furthermore, the measurement time of DNI was not uniform in the included studies^[8]^ and in some cases, it was not considered in the analysis (Table 1).^[9]^

**Table 1:**
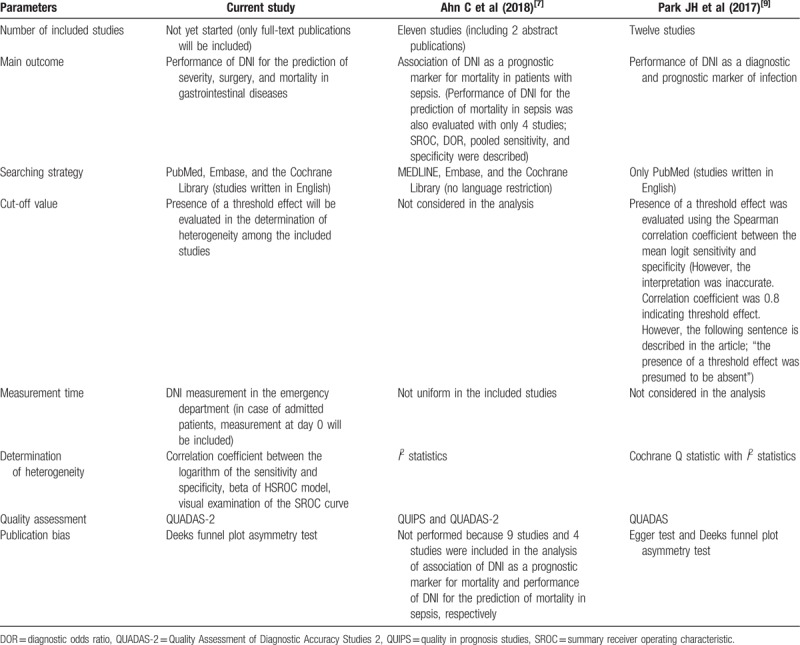
Comparison of previous meta-analyses with current study.

The pooled synthesis of the diagnostic performance of various gastrointestinal diseases with different cut-off values for DNI may limit the interpretation of uniform diagnostic validity. The authors will contact the corresponding authors for the missing values, requesting the original data in each study. However, if there are no responses from these authors, these studies will be excluded and this could be a potential bias in the meta-analysis.

These results of this study will provide diagnostic validity of DNI as an initial marker for the prediction of severity, surgery, and mortality of gastrointestinal diseases.

## Author contributions

**Conceptualization:** Chang Seok Bang

**Data curation:** Jae Ho Choi, Chang Seok Bang, Jae Jun Lee, Gwang Ho Baik

**Formal analysis:** Jae Ho Choi, Chang Seok Bang

**Funding acquisition:** Chang Seok Bang

**Investigation:** Jae Ho Choi, Chang Seok Bang, Jae Jun Lee, Gwang Ho Baik

**Methodology:** Chang Seok Bang

**Project administration:** Chang Seok Bang

**Resources:** Jae Ho Choi, Chang Seok Bang, Jae Jun Lee, Gwang Ho Baik

**Visualization:** Chang Seok Bang

**Writing – original draft:** Chang Seok Bang

**Writing – review & editing:** Chang Seok Bang

**Conceptualization:** Chang Seok Bang.

**Data curation:** Jae Ho Choi, Chang Seok Bang, Jae Jun Lee, Gwang Ho Baik.

**Formal analysis:** Jae Ho Choi, Chang Seok Bang.

**Funding acquisition:** Chang Seok Bang.

**Investigation:** Jae Ho Choi, Chang Seok Bang, Jae Jun Lee, Gwang Ho Baik.

**Methodology:** Chang Seok Bang.

**Project administration:** Chang Seok Bang.

**Resources:** Jae Ho Choi, Chang Seok Bang, Jae Jun Lee, Gwang Ho Baik.

**Visualization:** Chang Seok Bang.

**Writing – original draft:** Chang Seok Bang.

**Writing – review & editing:** Chang Seok Bang.

Chang Seok Bang orcid: 0000-0003-4908-5431.
